# Non‐Invasive Tumor Budding Evaluation and Correlation with Treatment Response in Bladder Cancer: A Multi‐Center Cohort Study

**DOI:** 10.1002/advs.202416161

**Published:** 2025-05-20

**Authors:** Xiaoyang Li, Chen Zou, Chunhui Wang, Cheng Chang, Yi Lin, Shuai Liang, Haoran Zheng, Libo Liu, Kai Deng, Lin Zhang, Bohao Liu, Mingchao Gao, Peicong Cai, Jianwen Lao, Longhao Xu, Daqin Wu, Xiao Zhao, Xiao Wu, Xinyuan Li, Yun Luo, Wenlong Zhong, Tianxin Lin

**Affiliations:** ^1^ Department of Urology Third Affiliated Hospital of Sun Yat‐sen University Sun Yat‐sen University 600th Tianhe Road Guangzhou 510630 P. R. China; ^2^ Department of Urology Sun Yat‐sen Memorial Hospital Sun Yat‐sen University Guangzhou 510120 P. R. China; ^3^ Department of Urology Yan'an Hospital Affiliated to Kunming Medical University Kunming Medical University Kunming 650051 P. R. China; ^4^ Department of Urology the Second Hospital of Dalian Medical University Dalian Medical University Dalian 116027 P. R. China; ^5^ Department of Urology Henan Provincial People's Hospital Zhengzhou 450003 P. R. China; ^6^ Department of Urology the First Affiliated Hospital of Chongqing Medical University Chongqing Medical University 1st Youyi Road Chongqing 400016 P. R. China

**Keywords:** bladder cancer, deep learning, multicenter study, neoadjuvant chemoimmunotherapy, tumor budding

## Abstract

The clinical benefits of neoadjuvant chemoimmunotherapy (NACI) are demonstrated in patients with bladder cancer (BCa); however, more than half fail to achieve a pathological complete response (pCR). This study utilizes multi‐center cohorts of 2322 patients with pathologically diagnosed BCa, collected between January 1, 2014, and December 31, 2023, to explore the correlation between tumor budding (TB) status and NACI response and disease prognosis. A deep learning model is developed to noninvasively evaluate TB status based on CT images. The deep learning model accurately predicts the TB status, with area under the curve values of 0.932 (95% confidence interval: 0.898–0.965) in the training cohort, 0.944 (0.897–0.991) in the internal validation cohort, 0.882 (0.832–0.933) in external validation cohort 1, 0.944 (0.908–0.981) in the external validation cohort 2, and 0.854 (0.739–0.970) in the NACI validation cohort. Patients predicted to have a high TB status exhibit a worse prognosis (*p* < 0.05) and a lower pCR rate of 25.9% (7/20) than those predicted to have a low TB status (pCR rate: 73.9% [17/23]; *p* < 0.001). Hence, this model may be a reliable, noninvasive tool for predicting TB status, aiding clinicians in prognosis assessment and NACI strategy formulation.

## Graphical abstract

This study introduces a deep learning model that predicts tumor budding (TB) status in bladder cancer through the analysis of CT images. The model effectively identifies patients with high TB status, correlating with poorer prognosis and reduced responsiveness to neoadjuvant chemoimmunotherapy. This tool offers significant potential to inform prognosis and enhance treatment decision‐making.

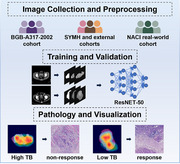

## 1 Introduction

Bladder cancer (BCa) represents the ninth most common malignancy worldwide, accounting for 3% of all cancer diagnoses globally.^[^ ^1^, ^2^ ^]^ Approximately 20–30% of patients present with muscle‐invasive bladder cancer (MIBC) at initial diagnosis. Cisplatin‐based neoadjuvant chemotherapy (NAC) followed by radical cystectomy (RC) is recommended as the standard treatment for MIBC.^[^ ^3^ ^]^ Nevertheless, more than 40% of patients experience disease progression within 3 years.^[^ ^4^, ^5^, ^6^ ^]^ Our recent multi‐institutional phase II trial and other studies have shown that neoadjuvant chemoimmunotherapy (NACI) may improve clinical outcomes, with a pathological complete response (pCR) rate of 33–50.9%.^[^ ^7^, ^8^, ^9^, ^10^ ^]^ Phase III studies suggest that NACI may significantly improve event‐free survival (EFS) and overall survival (OS) compared with NAC alone.^[^ ^11^ ^]^ Moreover, patients who achieve clinical CR after NACI may have the opportunity for bladder preservation, with a 2‐year OS rate of 97%.^[^ ^12^ ^]^ However, more than half of the patients do not achieve CR after NACI and may even develop metastasis, resulting in the loss of surgical options. This underscores the need for predictive models to assess treatment efficacy and guide personalized therapy.^[^ ^3^ ^]^

Tumor budding (TB), which is characterized by isolated single tumor cells or small clusters at the invasive front of a tumor, is significantly correlated with metastasis, poor prognosis, and treatment resistance in patients with various types of solid tumors.^[^ ^13^, ^14^, ^15^ ^]^ However, whether TB can act as a predictive biomarker of the response to neoadjuvant treatment remains unclear. Considering that TB is dynamic and changes with disease progression and treatment, performing repeated invasive procedures to monitor TB is impractical.^[^ ^16^ ^]^ Therefore, non‐invasive evaluation of TB status may be invaluable for longitudinal assessment.

Artificial intelligence (AI) can autonomously detect and analyze pixel‐level features and is increasingly used to assist precision medical and clinical decision support.^[^ ^17^ ^]^ AI‐based deep learning represents a promising approach for predicting the response to NAC in various cancers.^[^ ^18^ ^]^ Recent studies have demonstrated that several deep learning models leveraging non‐invasive computed tomography (CT) images have the potential to predict tumor mutations, immune checkpoint expression, and features of tumor microenvironment (TME), ultimately aiding in the prediction of NAC response.^[^ ^19^, ^20^, ^21^, ^22^, ^23^, ^24^ ^]^ However, there currently appears to be a lack of reports on TB‐based prediction models for solid tumors that utilize AI algorithms.

Herein, we integrated the clinicopathological information of patients with BCa from multi‐institutional cohorts and developed a deep‐learning model to assess TB status. We also validated the model's effectiveness in assessing NACI response and OS.

## 2 Result

### 2.1 Patient Cohort

The flowchart of this study is illustrated in Figure S1 (Supporting Information). Overall, 2322 patients met the inclusion criteria, of whom 2113 were enrolled in the study. The median age was 66 years (interquartile range [IQR]: 59.0–74.0), with 1724 men (81.6%) and 389 women (18.4%). Among the patients, 1170 (55.4%) were diagnosed with non‐muscle‐invasive bladder cancer (NMIBC), and 943 (44.6%) were diagnosed with MIBC. Additionally, 1343 patients (63.6%) were classified as low‐TB, whereas 770 (36.4%) were classified as high‐TB (Table S1, Supporting Information). Among the 2113 patients, 57 were from a multicenter, phase II clinical study constituting the BGB‐A317‐2002 cohort. Data on 514 patients from Sun Yat‐sen Memorial Hospital (SYMH) and 460 patients from Third Affiliated Hospital of Sun Yat‐sen University (SYUTH) pathologically diagnosed with BCa between January 1, 2014, and December 31, 2023, were retrospectively obtained, forming the SYMH cohort and external cohort 1, respectively. External cohort 2 comprised 647 patients from the Second Hospital of Dalian Medical University (SHDMU), First Affiliated Hospital of Chongqing Medical University (FHCMU), and Yan'an Hospital Affiliated to Kunming Medical University (KMYAYY) pathologically diagnosed with BCa between January 1, 2017 and December 31, 2023. We also retrospectively collected data from 108 patients from the five aforementioned hospitals in China who received NACI, constituting the NACI real‐world cohort. Tables S2–S4 (Supporting Information) show the detailed characteristics of all the cohorts.

**Figure 1:**
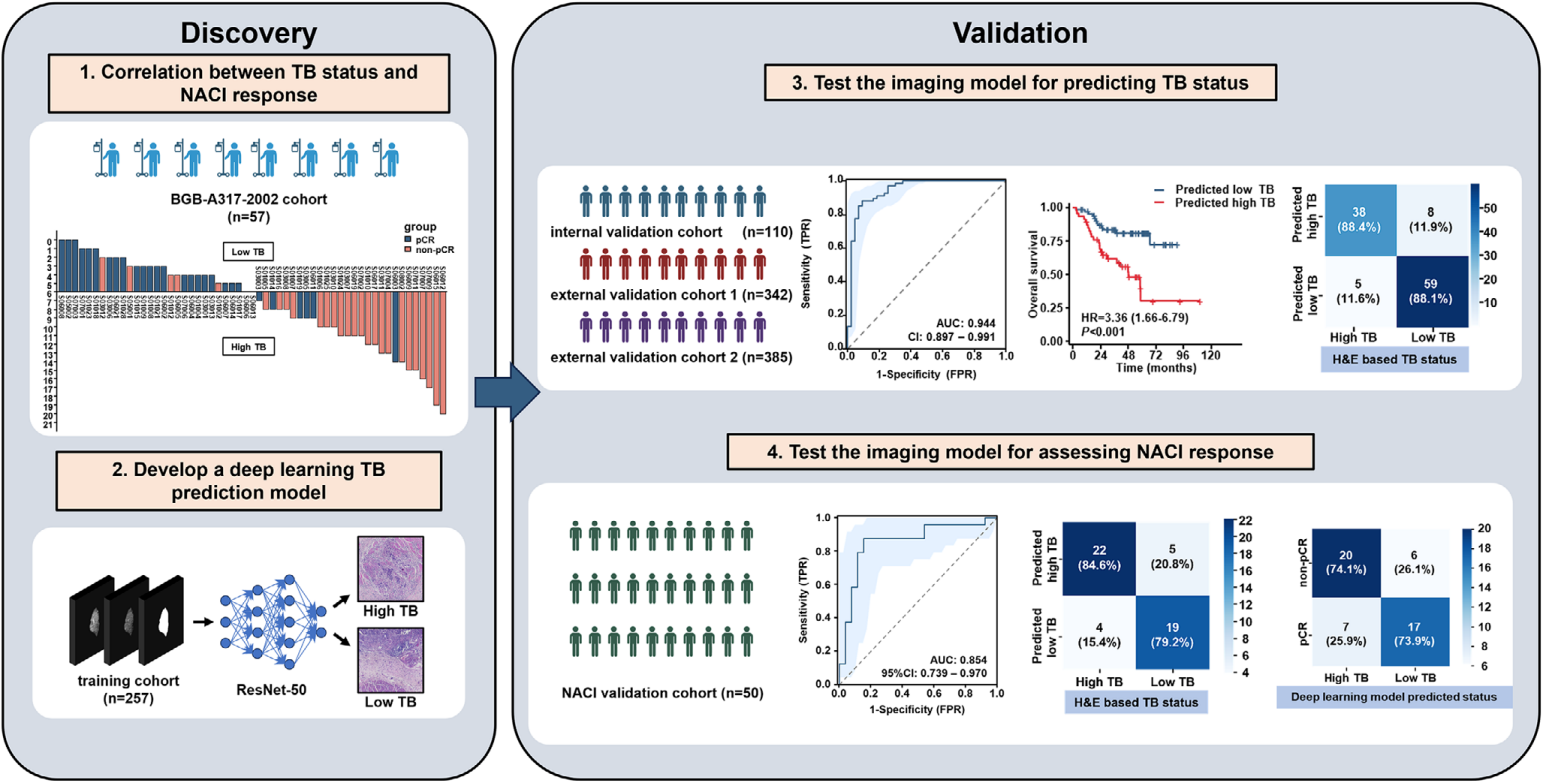
The overall workflow for the discovery and validation of a deep learning model based on CT images to predict the TB status and assess NACI response in patients with BCa. BCa, bladder cancer; CT, computed tomography; H&E, hematoxylin, and eosin; NACI, neoadjuvant chemoimmunotherapy; TB, tumor budding.

As illustrated in the workflow (**Figure** **1**), this study initially explored the correlation between TB status and response to NACI using data from the BGB‐A317‐2002 cohort. A deep learning‐based TB prediction model was then developed using the ResNet‐50 algorithm, with its performance validated in the internal validation cohort and external validation cohort 1 and 2. Finally, we assessed the evaluation capability of this AI‐based model for both prognosis and NACI response.

High‐quality preoperative contrast‐enhanced pelvic CT images from 1144 patients were included in the training and validation phases of the deep learning model. Among them, 367 patients in the SYMH cohort were assigned to the training (*n* = 257) and internal validation (*n* = 110) cohorts in a 7:3 ratio using a random number method. Additionally, 342 patients from external cohort 1 and 385 from external cohort 2 were grouped into external validation cohorts 1 and 2, respectively. Fifty patients from the NACI real‐world cohort constituted the NACI validation cohort, which was used to assess the ability of the model to assess NACI response.

### 2.2 TB Status is Associated with NACI Response and Dynamically Changes with NACI

**Figure 2:**
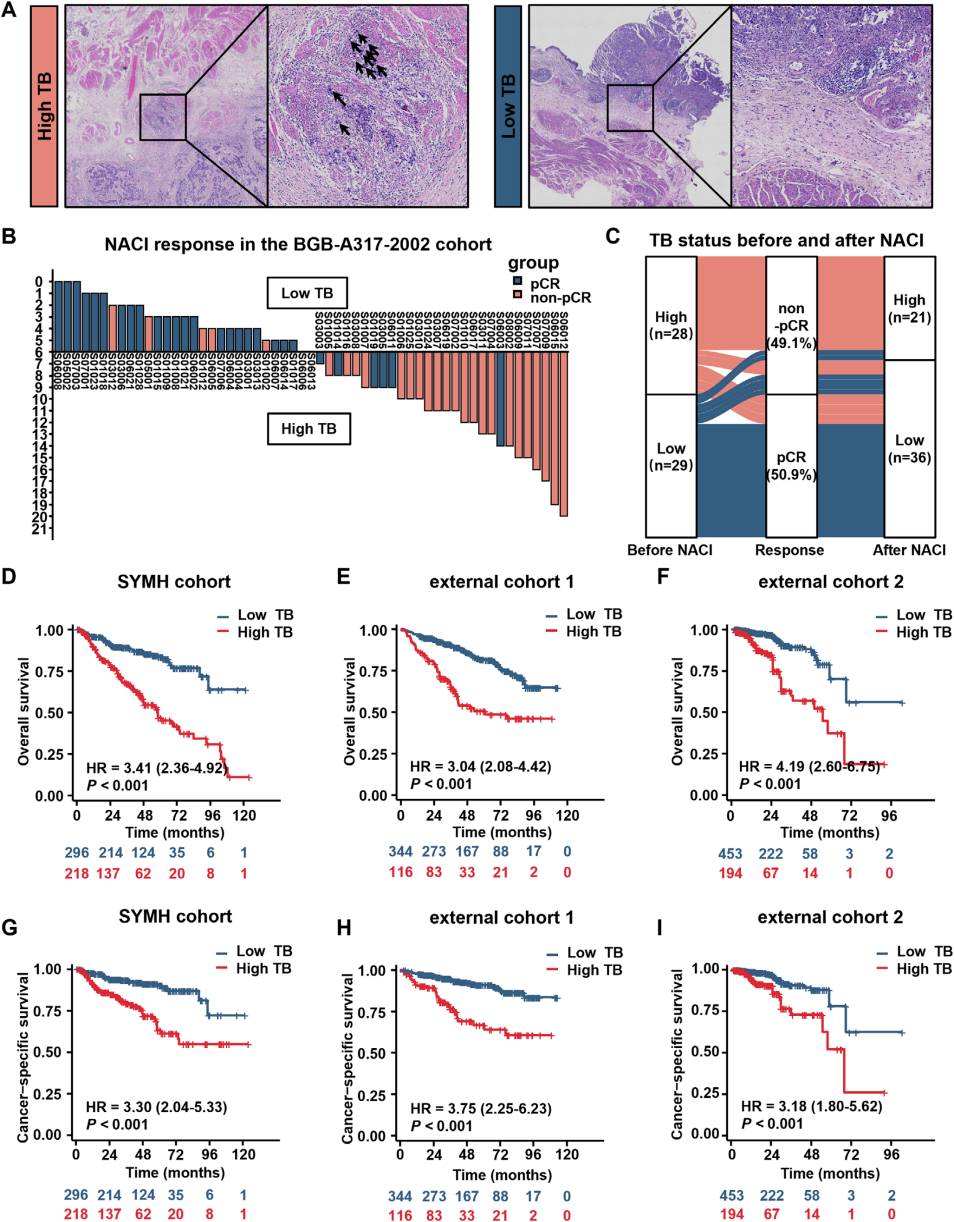
Correlation between TB status and NACI response and prognosis. A) Representative H&E‐stained slides showing high TB and low TB statuses (20×). B) The correlation between TB status and response to NACI in the BGB‐A317‐2002 cohort (*n* = 57; *p* < 0.001; Pearson's chi‐squared test). C) Changes in the TB status before and after NACI treatment in patients. D–I) Kaplan‐Meier analysis of overall and cancer‐specific survival of patients with BCa stratified by the TB status in the SYMH cohort (*n* = 514; *p* < 0.001), external cohort 1 (*n* = 460; *p* < 0.001), and external cohort 2 (*n* = 647; *p* < 0.001). *p*‐values were calculated using the Cox proportional hazards model. H&E, hematoxylin and eosin; HR, hazard ratio; K‐M curve, Kaplan‐Meier curve; NACI, neoadjuvant chemoimmunotherapy; pCR, pathological complete response; SYMH, Sun Yat‐sen Memorial Hospital; TB, tumor budding.

Initially, we analyzed the correlation between the TB status and NACI response in the BGB‐A317‐2002 cohort. Among the 57 patients, 86% (49/57) were men, with a median age of 64 years (IQR: 59.0‐68.0). All patients underwent NACI, and the pCR rate was 50.9% (29/57). Based on the analysis of the hematoxylin and eosin (H&E)‐stained slides, 29 patients (50.9%) were assigned to the low‐TB group, whereas 28 (49.1%) were assigned to the high‐TB group (Table S2, Supporting Information). Representative H&E‐stained slides of high‐ and low‐TB samples are shown in **Figure** **2**A. Among the low‐TB patients, 79.3% (23/29) achieved pCR, whereas only 21.4% (6/28) of the high‐TB patients achieved pCR (*p* < 0.001; Figure 2B), suggesting that patients with a high TB status were less likely to benefit from NACI than those with a low TB status.

Next, we evaluated the TB status of patients who underwent NACI and subsequent RC. The TB status changed significantly following treatment; 32.1% (9/28) of the patients with a high TB status before NACI showed a low TB status after NACI, and 67.7% (6/9) of these patients achieved pCR. Among the 29 patients with a low TB status before NACI, two transitioned to a high TB status, and none of them achieved pCR (Figure 2C). Changes in the TB status during NACI treatment significantly affected treatment outcomes and prognosis, further highlighting the importance of dynamic monitoring of TB status.

### 2.3 High TB is Correlated with Poor Prognostic Outcomes

We conducted a median follow‐up of 69.6 months (IQR: 49.0–88.7 months) and analyzed the relationship between TB status and survival outcomes. The OS and Cancer‐specific survival (CSS) rates in patients with a high TB status were significantly worse than those in patients with a low TB status in the SYMH cohort and external cohorts 1 and 2, with 5‐year OS rates of 49.95, 45.05, and 34.76% in the high‐TB group and 80.62, 80.44, and 74.29% in the low‐TB group, respectively (*p* < 0.05; Cox proportional hazards model; Figure 2D–I). The Cancer Genome Atlas (TCGA) and BGB‐A317‐2002 cohorts exhibited similar results (*p* < 0.001; Cox proportional hazards model; Figure S2a–e, Supporting Information). Univariate and multivariate Cox regression analyses of the combined cohort (1621 patients from the five hospitals) indicated that a high TB status was an independent risk factor for OS (HR: 0.487; 95% CI: 0.38–0.623; *p *< 0.001; Table S5, Supporting Information). Similar results were obtained for the individual cohorts (SYMH cohort and external cohorts 1 and 2; *p *< 0.001; Table S6–S8, Supporting Information).

To analyze the effect of TB status on survival outcomes, we examined its correlation with clinicopathological characteristics and then followed by subgroup analysis. In the SYMH cohort, a high TB status was correlated with an advanced T stage (muscular invasion), N status, grade, and likelihood of lymphovascular invasion (LVI), perineural invasion (PNI), and tumor multifocality (*p* < 0.05; Table S9, Supporting Information). Similar results were observed for external cohorts 1 and 2 and TCGA, BGB‐A317‐2002, and NACI real‐world cohorts (*p* < 0.05; Tables S9–S12, Supporting Information). For the subgroup analysis, hazard ratios (HRs) from the Cox proportional hazards model comparing high‐ and low‐TB subgroups based on sex, age, T stage, N status, grade, LVI, PNI, carcinoma in situ (CIS), and tumor multifocality are presented in a forest plot. Except for the women (*p* = 0.097), concomitant CIS (*p* = 0.911), and PNI (*p* = 0.067) subgroups, high TB status appears to negatively affect OS in all other subgroups (*p* < 0.05, Figure S3, Supporting Information). These results demonstrated the accuracy of the prognostic prediction of TB status, independent of other clinicopathological characteristics.

### 2.4 The Deep Learning Model Demonstrates Robust Predictive Performance

**Figure 3:**
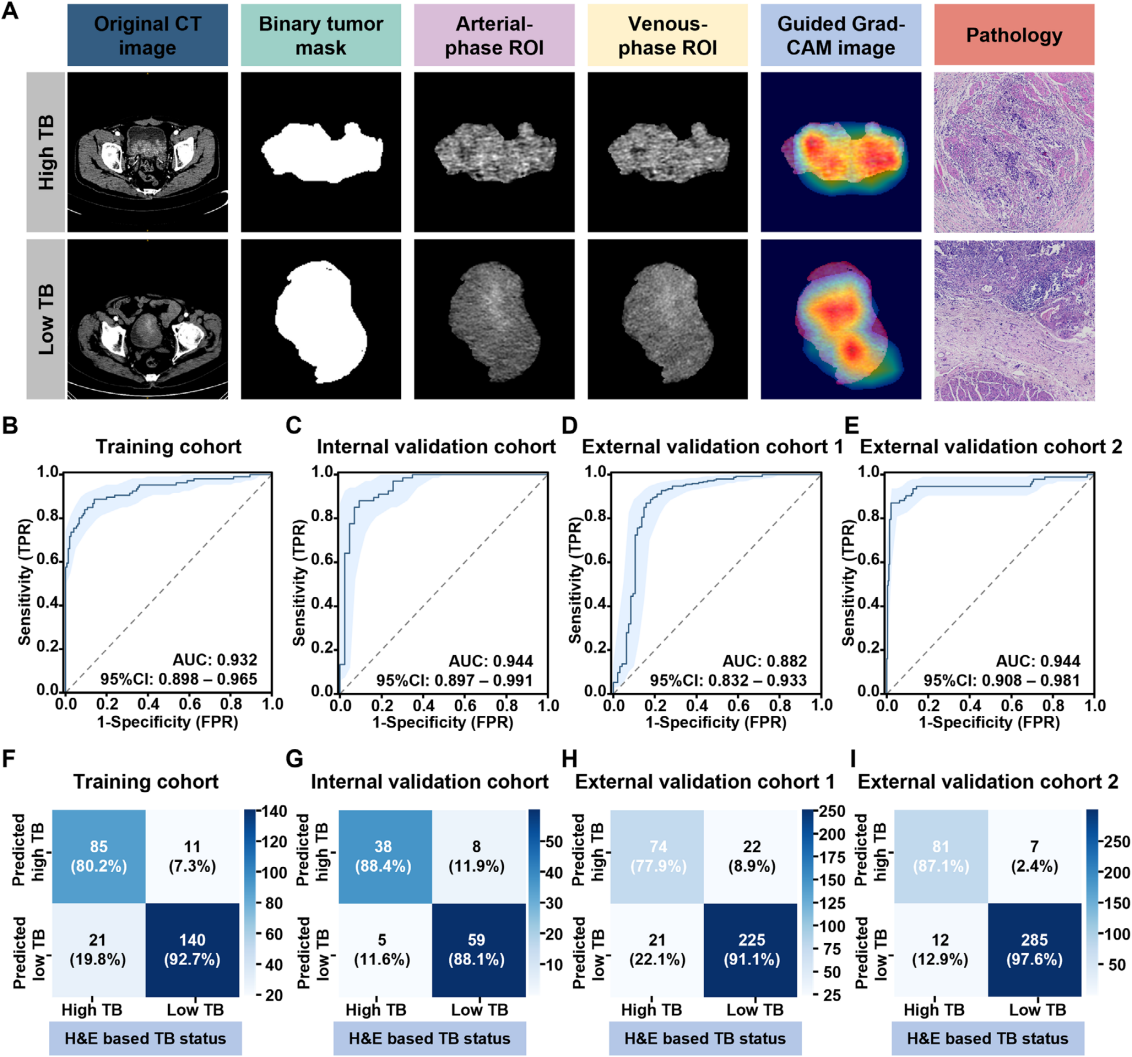
Performance of the TB‐based deep learning prediction model across different cohorts. A) Representative examples of the original CT images showing the three input channels: binary tumor mask, arterial‐phase ROI, and venous‐phase ROI. The model prediction is visualized using Guided Grad‐CAM, along with the corresponding pathological images. B–E) AUCs and 95% CIs of results obtained using the deep learning TB prediction model for the training cohort (*n* = 257), internal validation cohort (*n* = 110), external validation cohort 1 (*n* = 342), and external validation cohort 2 (*n* = 385). F–I) Sensitivity and specificity of results obtained using the TB‐based deep learning prediction model for the training cohort (*n* = 257), internal validation cohort (*n* = 110), external validation cohort 1 (*n* = 342), and external validation cohort 2 (*n* = 385). AUC, the area under the curve; 95% CI, 95% confidence interval; CT, computed tomography; Guided Grad‐CAM, guided gradient‐weighted class activation mapping; H&E, hematoxylin, and eosin; ROI, region of interest; TB, tumor budding.

Giving the significant impact of TB on the NACI response and prognosis, as well as its dynamic nature, we developed an AI‐based non‐invasive model for predicting TB status using CT images. Representative examples of the original CT images, the three input channels (binary tumor mask, arterial‐phase region of interest [ROI], and venous‐phase ROI), visualization using guided gradient‐weighted class activation mapping approach (Guided Grad‐CAM), and H&E assessment of the TB status are presented in **Figure** **3**A. For the training cohort, the model achieved an area under the curve (AUC) of 0.932 (95% CI: 0.898–0.965) for predicting TB status (Figure 3B). Similarly, the model attained AUCs of 0.944 (0.897–0.991), 0.882 (0.832–0.933), and 0.944 (0.908–0.981) for the internal and external validation cohorts 1 and 2, respectively (Figure 3C–E). The overall accuracies were 87.55, 88.18, 87.43, and 95.06% for the training, internal, and external validation cohorts 1 and 2, respectively. The sensitivity, specificity, positive predictive value (PPV), and negative predictive value (NPV) were 0.884 (0.824‐0.944), 0.881 (0.82‐0.941), 0.826 (0.755‐0.897), and 0.922 (0.872‐0.972), respectively, for the internal validation cohort (Figure 3F–I and **Table** **1**). Similarly, the model demonstrated robust performance regarding the results for the other cohorts and exhibited high discriminative power (Figure 3F–I and Table 1).

**Table 1:** Performance of the TB‐based deep learning prediction model in the training and validation cohorts.

	Over accuracy	AUC (95% CI)	Sensitivity (95% CI)	Specificity (95% Cl)	PPV (95% Cl)	NPV (95% Cl)
Training cohort	87.55%	0.932 (0.898–0.965)	0.802 (0.753–0.851)	0.927 (0.895–0.959)	0.885 (0.847–0.924)	0.870 (0.828–0.911)
Internal validation cohort	88.18%	0.944 (0.897–0.991)	0.884 (0.824–0.944)	0.881 (0.820–0.941)	0.826 (0.755–0.897)	0.922 (0.872–0.972)
External validation cohort 1	87.43%	0.882 (0.832–0.933)	0.779 (0.735–0.823)	0.911 (0.881–0.941)	0.771 (0.726–0.815)	0.915 (0.885–0.944)
External validation cohort 2	95.06%	0.944 (0.908–0.981)	0.871 (0.838–0.905)	0.976 (0.961–0.991)	0.920 (0.893–0.948)	0.960 (0.940–0.979)

AUC, area under the curve; 95% CI, 95% confidence interval; NPV, negative predictive value; PPV, positive predictive value; TB, tumor budding.

**Figure 4:**
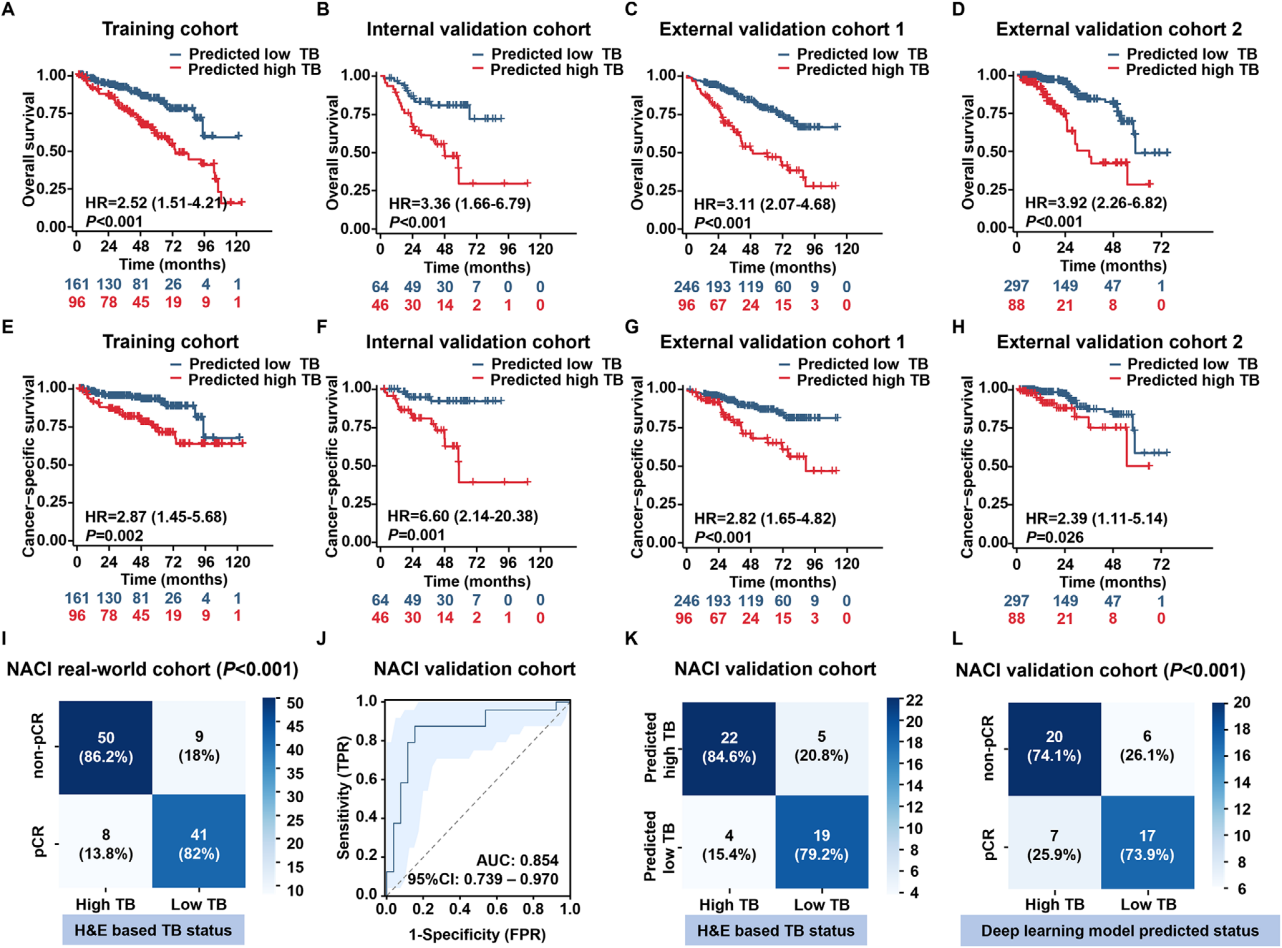
Evaluation ability of the model for prognosis and response to NACI. A–D) Kaplan‐Meier analysis of the overall survival of patients with BCa stratified by the predicted TB status in the training cohort (*n* = 257; *p* < 0.001), internal validation cohort (*n* = 110; *p* < 0.001), external validation cohort 1 (*n* = 342; *p* < 0.001), and external validation cohort 2 (*n* = 385; *p* < 0.001). *P*‐values were calculated using the Cox proportional hazards model. E–H) Kaplan‐Meier analysis of the cancer‐specific survival of patients with BCa stratified by the predicted TB status in the training cohort (*n* = 257; *p* = 0.002), internal validation cohort (*n* = 110; *p* < 0.001), external validation cohort 1 (*n* = 342; *p* < 0.001), and external validation cohort 2 (*n* = 385; *p* = 0.026). *p*‐values were calculated using the Cox proportional hazards model. I) H&E‐based TB status and response to NACI in the NACI real‐world cohort (*n* = 108; *p* < 0.001; Pearson's chi‐squared test). J) AUCs and 95% CIs of results obtained using the TB‐based deep learning prediction model for the NACI validation cohort (*n* = 50). K) Sensitivity and specificity of results obtained using the TB‐based deep learning prediction model for the NACI validation cohort (*n* = 50). L) Predicted TB status and response to NACI in the NACI validation cohort (*n* = 50; *p* < 0.001; Pearson's chi‐squared test). AUC, the area under the curve; BCa, bladder cancer; 95% CI, 95% confidence interval; H&E, hematoxylin, and eosin; HR, hazard ratio; NACI, neoadjuvant chemoimmunotherapy; pCR, pathological complete response; TB, tumor budding.

The model also demonstrated a strong ability to assess prognosis. Patients predicted to have high TB status exhibited significantly worse OS and CSS rates compared with those predicted to have low TB status across all cohorts (*p *< 0.05; Cox proportional hazards model; **Figure** **4**A–H). Multivariate time‐independent Cox regression analysis yielded similar results (Table S13, Supporting Information).

### 2.5 The Deep Learning Model Effectively Evaluates the NACI Response by Predicting TB Status

To validate the ability of the model to assess the NACI response, we retrospectively collected data from the NACI real‐world cohort (*n* = 108). The data distribution was similar to that of the BGB‐A317‐2002 cohort, in which 46.3% (50/108) of patients were placed in the low‐TB category, of whom 82% (41/50) achieved pCR. In contrast, only 13.8% (8/58) of high‐TB patients reached pCR (*p* < 0.001; Pearson's chi‐squared test; Figure 4I; Table S11, Supporting Information). The deep learning model demonstrated high evaluation performance in the NACI validation cohort (AUC: 0.854; 95% CI: 0.739‐0.97; Figure 4J), with a sensitivity of 84.6% (22/26) and specificity of 79.2% (19/24; Figure 4K). Notably, the prediction model achieved a pCR rate of 73.9% (17/23) in patients predicted to have a low TB status, whereas 74.1% (20/27) of those predicted to have a high TB status did not achieve pCR (*p* < 0.001; Pearson's chi‐squared test; Figure 4L), demonstrating the potential ability of the model in guiding clinical decision‐making.

## 3 Discussion

In this multicenter cohort study, we comprehensively analyzed the association between TB and the response to NACI, and demonstrated the high prognostic value of TB in cases of BCa. Subsequently, we developed and validated a deep learning model to non‐invasively assess the TB status using CT images. We demonstrated that the model may have significant implications for the selection of diagnostic and therapeutic strategies.

Although TB was initially proposed as a prognostic factor for colorectal cancer (CRC), an increasing number of studies have suggested that it is also a crucial factor in the prognosis and therapeutic outcomes of breast, pancreatic, and lung cancers, as well as other solid tumors.^[^ ^16^ ^]^ Consistently, this was validated across multiple cohorts of BCa in the current study. Furthermore, the TB prediction model we developed may have valuable applications for disease evaluation. In this study, patients predicted to have a high TB status may have more aggressive disease and a poorer prognosis than those predicted to have a low TB status.

Biologically, TB is closely associated with epithelial‐mesenchymal transition (EMT) and immune evasion. E‐cadherin, serving as a cell‐cell adhesion molecule, is observed to be downregulated or lost on the surface of tumor buds, leading to the dissociation of tumor cells.^[^ ^16^ ^]^ Cytoskeletal changes and increased proteolytic activity in tumor buds facilitate cell migration.^[^ ^25^, ^26^ ^]^ TB may also affect the efficacies of immunotherapy and chemotherapy.^[^ ^27^, ^28^ ^]^ Tumor cells undergoing EMT show elevated programmed death‐ligand 1 (PD‐L1) levels, which suppresses cytotoxic T‐cell attacks, leading to immune evasion and decreased sensitivity to immune checkpoint inhibitors.^[^ ^29^ ^]^ Viktor et al. found that tumor buds at the invasive front can achieve immune evasion by downregulating major histocompatibility complex‐I (MHC‐I) molecules on the cell membrane.^[^ ^30^ ^]^ These studies have provided molecular‐level insights into the correlation between TB and the NACI response. Herein, patients with a high TB status were unlikely to benefit from NACI (only 21.4% in the BGB‐A317‐2002 cohort and 13.8% in the NACI real‐world cohort), further supporting this notion.

The TB status changed dynamically during NACI and was closely associated with treatment outcomes. However, it was assessed using postoperative pathological H&E‐stained slides, posing a challenge for the real‐time monitoring of TB status to guide treatment decisions. We addressed this issue using a deep learning convolutional neural network (CNN) based on CT images, offering an innovative solution. The model may be a reliable tool for clinicians in their decision‐making processes. For patients with predicted low TB status, NACI presents a viable treatment option, significantly increasing the likelihood of achieving pCR. Additionally, real‐time monitoring of TB status during NACI is crucial. A transition from low to high TB status may indicate diminished benefits from continued NACI, suggesting that more aggressive interventions, such as RC, may be warranted.

Many studies have integrated CNNs with imaging modalities such as CT, radiography, and magnetic resonance imaging to directly predict treatment outcomes or prognosis.^[^ ^31^, ^32^, ^33^ ^]^ In comparison with previous studies, our model encompasses several advancements. First, our study builds upon substantial evidence supporting the established link between TB status and disease prognosis, as well as its impact on treatment response. This foundation provides a biological basis for the model's predictions and addresses a critical issue of biological interpretability that has often been absent. Second, whereas most studies used images from a single phase, we incorporated both arterial‐ and venous‐phase contrast‐enhanced CT images to train the model. By integrating features from different phases, the model achieved enhanced generalization capability and reduced overfitting.^[^ ^20^ ^]^ Third, we built the model using a pre‐trained ResNet‐50 framework, which addressed the issues of vanishing and exploding gradients in deep neural network training by introducing residual blocks. By stacking 50 residual blocks, complex features were captured. This led to outstanding prediction accuracy and generalization ability, with high robustness and low error rates.

The current study had a few limitations. First, the TB prediction model was based on retrospectively collected imaging data, which, despite the implementation of quality control and assurance measures, remained susceptible to significant bias. Second, the prediction model required manual annotation of ROI, which introduces bias and poses challenges for routine clinical use. Third, due to the relatively small sample size of patients undergoing NACI, the prediction model does not provide direct predictions (Figure S4 and Table S14, Supporting Information) but instead assesses the correlation between tumor burden (TB) status and treatment response. Future studies with larger cohorts of patients undergoing NACI are necessary to validate and expand these findings.

In summary, we explored the positive correlations among TB, NACI resistance, and disease prognosis. Our prediction model, developed using preoperative contrast‐enhanced CT images, may enable the non‐invasive prediction of TB status in patients with BCa. This model can improve the accuracy of prognostic predictions and guide treatment decisions in patients with MIBC who require NACI. Prospective validation and pan‐cancer analyses are required to confirm the reproducibility and generalizability of the model in a broader patient population.

## 4 Experimental Section

### Patients

This multicenter cohort study was conducted across a phase II multicenter clinical trial (trial number: ChiCTR2000037670), five hospitals in China (Figure S5, Supporting Information), and TCGA database (https://portal.gdc.cancer.gov). The inclusion criteria included a histologically confirmed diagnosis of BCa (including NMIBC and MIBC) and the availability of clinicopathological and follow‐up data. Patients with other concurrent malignancies or a history of NAC treatment were excluded.

Patient demographics, pathological examination results, and H&E slides were obtained. Sex was reported as “sex assigned at birth.” Cancer staging was conducted based on the criteria outlined in the eighth edition of the American Joint Committee on Cancer TNM Staging Manual.^[^ ^34^ ^]^ For patients in the TCGA datasets, high‐quality H&E images suitable for TB status evaluation were obtained; however, data on neoadjuvant treatment were unavailable. The surgical options (transurethral resection of bladder tumor [TURBT] or RC) and follow‐up protocols were conducted in accordance with guidelines of the European Association of Urology (EAU) and the Chinese Urological Association (CUA).^[^ ^35^, ^36^ ^]^ Patients with TURBT were monitored every 3–6 months during the first 2 years, primarily through cystoscopies and urinary cytology. For patients with RC, follow‐up was performed at 3, 6, 12, 18, and 24 months after surgery, including physical examinations, routine laboratory panels, and CT of the chest, abdomen, and pelvis. OS was defined as the period from surgery to death from any cause. CSS was defined as the period from surgery until death attributable to cancer. The NACI response was evaluated using the pCR, which was defined as the achievement of a pathological stage of pT0N0 after RC.

Following the analysis of the correlation between TB and the NACI response, as well as the prognosis of all included patients, we developed a TB prediction model using preoperative contrast‐enhanced pelvic CT images. Patients were excluded if they lacked preoperative contrast‐enhanced pelvic CT images, or if their primary tumors could not be clearly identified due to factors such as poor bladder filling, large blood clots, or metallic artifacts from joint replacements or intrauterine devices.

This study was approved by the Ethics Committees of SYUTH (approval number: II2023‐303‐02), SYMH (approval number: SYSKY2023‐467‐01), SHDMU (approval number: 2022–141), FHCMU (approval number: 2022‐K508), and KMYAYY (approval number: 2024‐148‐01). Informed consent was acquired from all patients. This investigation was carried out in strict adherence to the ethical standards set forth in the Declaration of Helsinki.

### Assessment of TB

The method for assessing TB status was adapted from the standardized protocol recommended by the 2016 International Tumor Budding Consensus Conference and modified to accommodate the unique characteristics of BCa.^[^ ^37^ ^]^ Briefly, the H&E‐stained section with the deepest tumor invasion was selected, and a whole slide image was generated by scanning it at 20× magnification. Ten individual fields were selected to identify the field with the highest density of tumor buds (hotspots) at the invasive front. Tumor buds were counted in the selected “hotspot” (20× objective), and the number of buds was divided by the normalization factor to determine the tumor bud count per 0.785 mm^2^.

Considering that the current standards for classifying TB are primarily based on the guidelines for CRC and that the classification criteria for BCa remain unclear, we believe that simply adopting the CRC standards for BCa would not yield accurate results as CRC and BCa are tumors with different histological origins.^[^ ^34^ ^]^ Therefore, we utilized the data obtained by us to determine the optimal cutoff value for TB based on prognostic outcomes, which was calculated to be 6. Coincidentally, this cutoff value aligns with those selected in two other related studies that included 621 and 108 patients with BCa, respectively, further supporting the reliability of our cutoff value.^[^ ^38^, ^39^ ^]^

Dual‐independent assessments were performed by two investigators blinded to all clinical parameters to evaluate observer concordance. The classification concordance was 90% (Cohen's kappa = 0.867).

### CT Image Acquisition and Processing

All patients, whose data were analyzed using the deep learning model, underwent contrast‐enhanced pelvic CT prior to surgery. All CT sequences were obtained from each hospital and standardized in Neuroimaging Informatics Technology Initiative (NIfTI) format for further processing.

To comply with the model's computational specifications, CT images underwent preprocessing through bilinear interpolation resampling to 256 × 256 pixel resolution. Image intensities were normalized to a standardized Hounsfield unit (HU) range of −25 to 175 to enhance soft‐tissue contrast. The ROI was defined as the tumor mass and its basal region, with a focus on the anterior margin of tumor invasion. Consistent with previous studies, we selected the CT axial slice displaying the largest tumor base for manual ROI segmentation and labeling.^[^ ^19^, ^40^ ^]^ Efforts were made to avoid necrotic areas, calcifications, and large vessels whenever possible.

The ROIs were delineated using ITK‐SNAP (version 4.0.0) by two urological radiologists: one with more than 10 years and the other with over 30 years of experience in pelvic CT interpretation. Both radiologists were blinded to the clinical and pathological findings.

### Development of the Prediction Model

The TB prediction model was developed using the ResNet‐50 framework pre‐trained on ImageNet, which employs residual blocks and skip connections to mitigate challenges such as vanishing gradients and model degradation. We modified the model by replacing the original fully connected layer with a linear layer (torch.nn.Linear) containing a single output node, enabling the model to produce a single prediction value. The loss function used was BCEWithLogitsLoss for binary classification, and the Adam optimizer with weight decay for L2 regularization was employed to prevent overfitting. The structure of the modified model is shown in Figure S6 (Supporting Information). The input image consisted of three 150 × 150 pixels channels: the binary tumor mask, derived from the ROI, provides fundamental anatomical delineation, while the arterial‐ and venous‐phase ROI, segmented using the tumor mask, offers unique functional and morphological information for comprehensive tumor characterization.

The deep learning model was developed using the PyTorch framework (version 2.2.2+cu121) on a GeForce RTX 3080Ti GPU. The batch size was set to 64, with an initial learning rate of 0.0005 and a decay rate of 0.1 per 50 epochs. The training was conducted for a maximum of 2000 epochs, with early stopping triggered after 50 consecutive epochs without improvement.

A Guided Grad‐CAM approach was used to identify and visualize the areas within the ROI that contributed most to the prediction. All relevant code was executed using Visual Studio Code (VS Code, version 1.60.0) with Python (version 3.11.7) and can be accessed at https://github.com/854544429/load_dataset.git.

### Accuracy of the Imaging Model in Predicting TB Status

Performance indicators, such as overall accuracy, AUC, sensitivity, specificity, PPV, and NPV, were evaluated. The formulae for these metrics are provided in the method section of the Supporting Information.

### Statistical Analysis

Descriptive statistics were used to summarize the baseline characteristics. Continuous variables are presented as medians with IQR when they are skewed. Categorical variables are presented as frequencies and percentages. Continuous variables were compared using the Mann–Whitney U test or the Kruskal–Wallis test. Differences in categorical variables were evaluated using Pearson's chi‐squared test or Fisher's exact test. The correlation analysis between TB and prognosis was performed using the Kaplan‐Meier method and the Cox proportional hazards model. Schoenfeld residuals were used to evaluate the proportional hazard (PH) assumption for each variable. For variables that violated the PH assumption, multiplicative interaction terms were incorporated into the time‐dependent Cox regression analysis for OS and CSS. Relative risk was assessed by calculating HRs with 95% confidence intervals (95% CIs). Variables with prognostic significance in the univariate Cox regression analysis (*p *< 0.1) were incorporated into the multivariate analysis. The results of the subgroup analysis are presented using forest plots. The 95% CIs were calculated using the Clopper‐Pearson method. R software (version 4.3.1) was used for statistical analyses. A two‐sided *p*‐value of less than 0.05 was considered statistically significant.

## Conflict of Interest

The authors declare no conflict of interest.

## Author Contributions

X.L., C.Z, C.W, and C.C. contributed equally to this work. T.L. and W.Z. proposed the hypothesis and designed the study. X.L. and C.Z. designed the study and conducted the data analysis and manuscript writing. C.W. and C.C. contributed to data analysis and manuscript revision. Y.L., S.L., and H.Z. performed the H&E staining of the pathological slides. L.L., K.D., L.Z., and B.L. collected clinical data and conducted the slide scanning. M.G., P.C., and J.L. collected samples and additional clinical data. L.X., D.W., X.Z., and X.W. conducted the patient follow‐ups. X.L. and Y.L. modified and revised the manuscript; X.L., Y.L., W.Z., and T.L. supervised the study design and finalized the manuscript. All authors reviewed the manuscript, approved the submitted version, had full access to all the data reported in the study, and had final responsibility for the decision to submit the manuscript for publication.

## Supporting information



Supporting Information

## Data Availability

The data that support the findings of this study are available on request from the corresponding author. The data are not publicly available due to privacy or ethical restrictions.
