# Supplementary material for: Non‐Invasive Tumor Budding Evaluation and Correlation with Treatment Response in Bladder Cancer: A Multi‐Center Cohort Study
Source: Adv Sci (Weinh). 2025 May 20;12(22):2416161. doi: 10.1002/advs.202416161 (PMC12165028; doi:10.1002/advs.202416161)
Supplement: Supplementary file 1 — Supporting Information [file ADVS-12-2416161-s001.docx]

Supporting Information

Non-invasive Tumor Budding Evaluation and Correlation with Treatment Response in Bladder Cancer: A Multi-center Cohort Study

*Xiaoyang Li, Chen Zou, Chunhui Wang, Cheng Chang, Yi Lin, Shuai Liang, Haoran Zheng, Libo Liu, Kai Deng, Lin Zhang, Bohao Liu, Mingchao Gao, Peicong Cai, Jianwen Lao, Longhao Xu, Daqin Wu, Xiao Zhao, Xiao Wu, Xinyuan Li*, Yun Luo*, Wenlong Zhong*, Tianxin Lin**

**e****Methods**

**Figure S1.** Study flowchart.

**Figure S2.** Prognostic value of the TB status in the TCGA and BGB-A317-2002 cohorts.

**Figure S3.** Subgroup analysis of overall survival in the combined cohort.

**Figure S4.** AUCs and CIs of results obtained using the NACI response prediction model in patients underwent NACI.

**Figure S5.** Geographic distribution of the included hospitals in China and the number of patients enrolled from each hospital.

**Figure S6.** Architecture of the deep learning model (ResNet-50) used for tumour budding prediction.

**Table S1.** Clinicopathological characteristics of patients in the multi-institutional cohorts.

**Table S2.** Clinicopathological characteristics of patients in the BGB-A317-2002 cohort.

**Table S3.** Clinicopathological characteristics of patients in the SYMH and external cohorts.

**Table S4.** Clinicopathological characteristics of patients in the TCGA and NACI real-world cohorts.

**Table S5.** Time-dependent Cox regression analysis of the association between TB and OS in the combined cohort.

**Table S6.** Time-dependent Cox regression analysis of the association between TB and OS in the SYMH cohort.

**Table S7.** Time-dependent Cox regression analysis of the association between TB and OS in external cohort 1.

**Table S8.** Time-dependent Cox regression analysis of the association between TB and OS in external cohort 2.

**Table S9.** Clinicopathological characteristics of patients in the high- and low-TB groups in the SYMH and external cohorts.

**Table S10.** Clinicopathological characteristics of patients in the high- and low-TB groups in the TCGA cohort.

**Table S11.** Clinicopathological characteristics of patients in the high- and low-TB groups in the BGB-A317-2002 cohort.

**Table S12.** Clinicopathological characteristics of patients in the high- and low-TB groups in the NACI real-world cohort.

**Table S13.** Time-dependent Cox regression analysis of the association between the predicted TB status and OS in the combined cohort.

**Table S14.** Performance of the NACI response prediction model in patients underwent NACI.

**eMethods**

**the formulae for accuracy, sensitivity, specificity, positive predictive value (PPV), and negative predictive value (NPV)**

|  | **Predicted Positive** | **Predicted Negative** |
| --- | --- | --- |
| **Actual Positive** | True Positive (TP) | False Negative (FN) |
| **Actual Negative** | False Positive (FP) | True Negative (TN) |

Accuracy = (TP+TN)/(TP+TN+FP+FN)

Sensitivity = TP/(TP+FN)

Specificity = TN/(TN+FP)

Positive Predictive Value (PPV) = TP/(TP+FP)

Negative Predictive Value (NPV) = TN/(TN+FN)

**Supplemental Figures**


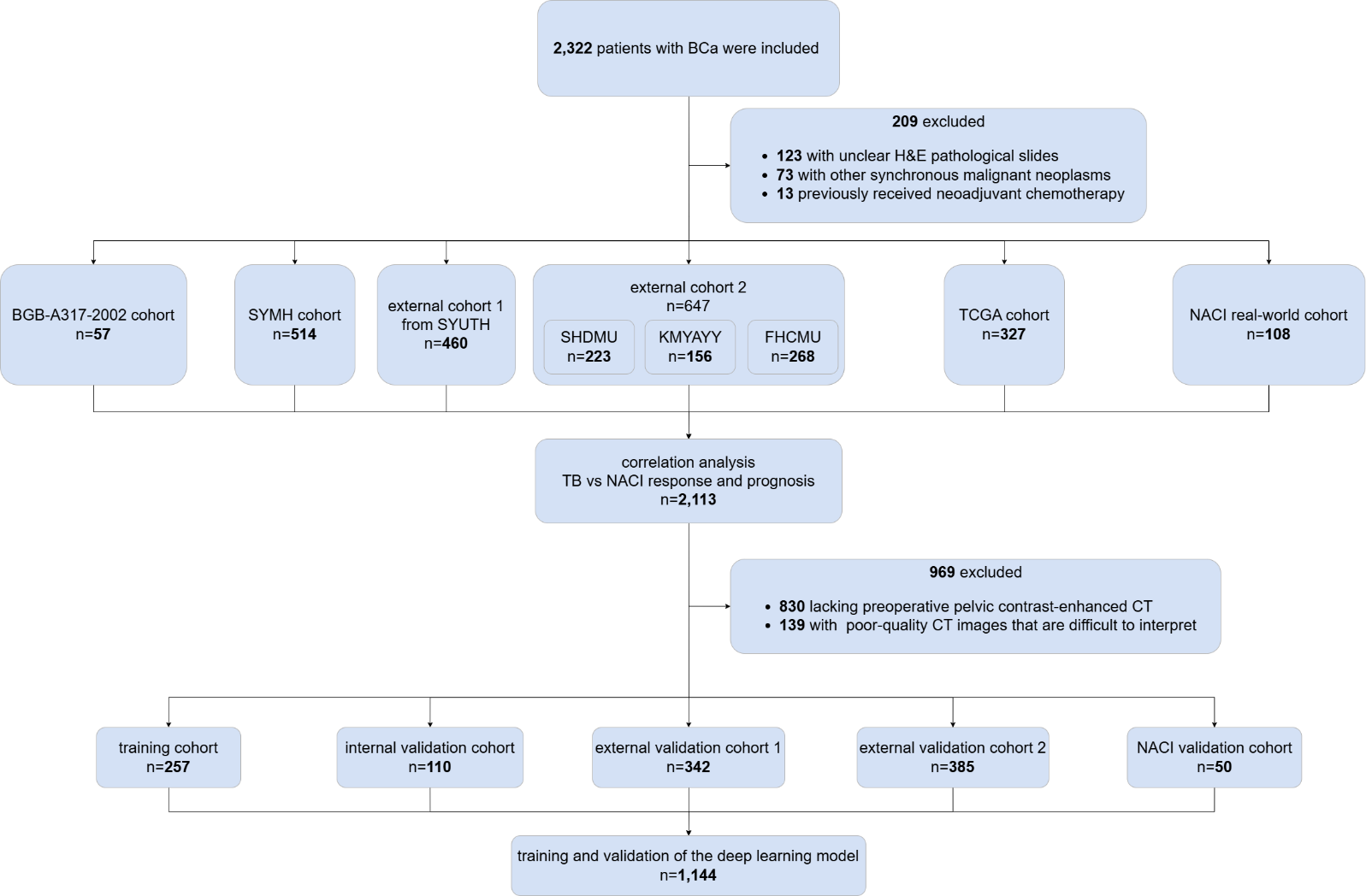


**Figure S1. Study flowchart.**

BCa: bladder cancer; CT: computed tomography; FHCMU: First Affiliated Hospital of Chongqing Medical University; H&E: hematoxylin and eosin; KMYAYY: Yan’an Hospital Affiliated to Kunming Medical University; NACI: neoadjuvant chemoimmunotherapy; SYMH: Sun Yat-sen Memorial Hospital; SYUTH: Third Affiliated Hospital of Sun Yat-sen University; SHDMU: Second Hospital of Dalian Medical University; TCGA: The Cancer Genome Atlas; TB: tumour budding.

**
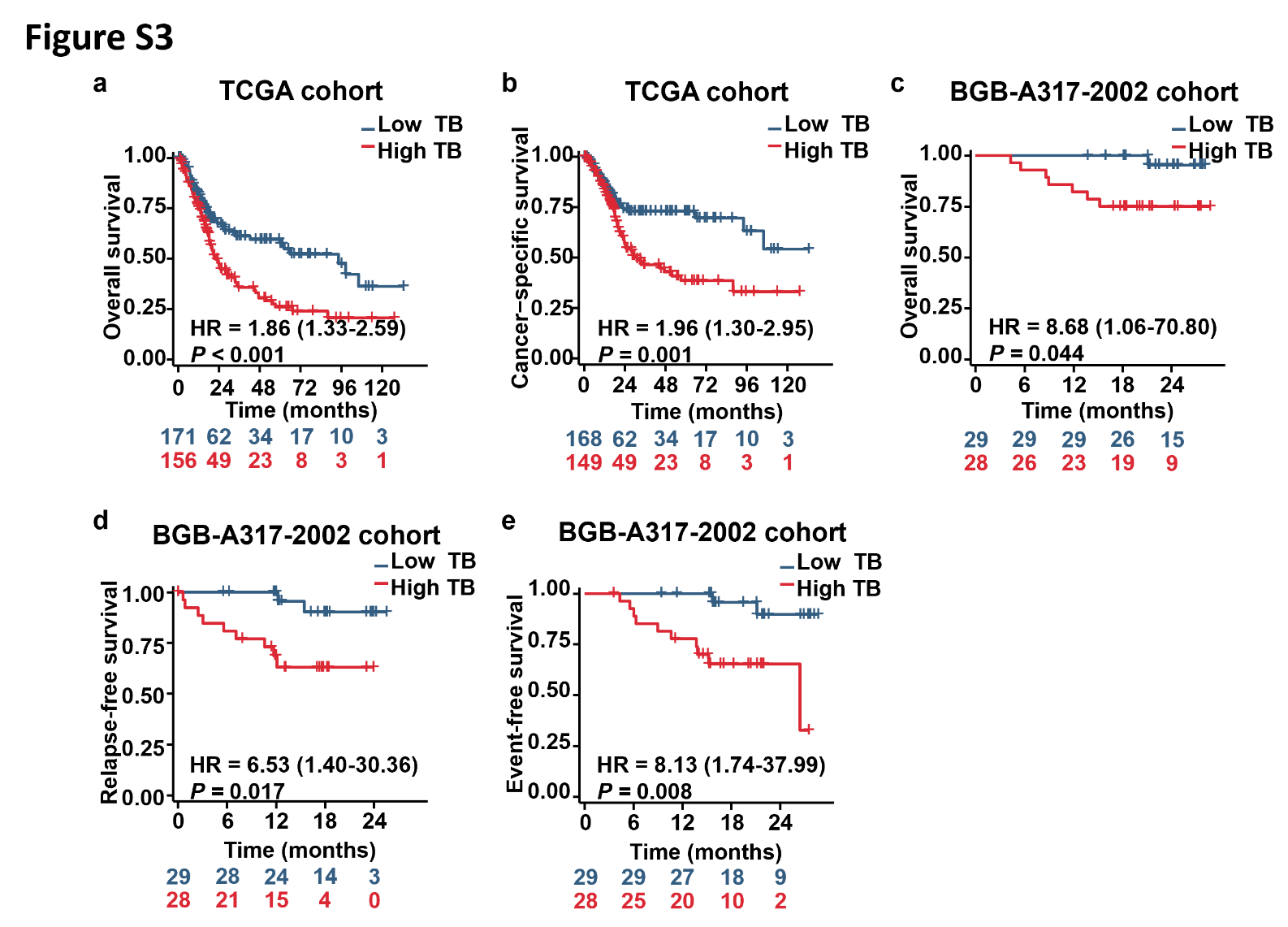
**

**Figure S2. Prognostic value of the TB status in the TCGA and BGB-A317-2002 cohorts.**

(a-e) Kaplan-Meier analysis of cancer-specific, overall, relapse-free, and event-free survival of patients with bladder cancer (BCa) stratified by the TB status in the TCGA cohort (n = 327) and BGB-A317-2002 cohort (n = 57). *P*-values were calculated using the Cox proportional hazards model. HR, hazard ratio; TB, tumour budding; TCGA, The Cancer Genome Atlas.

**
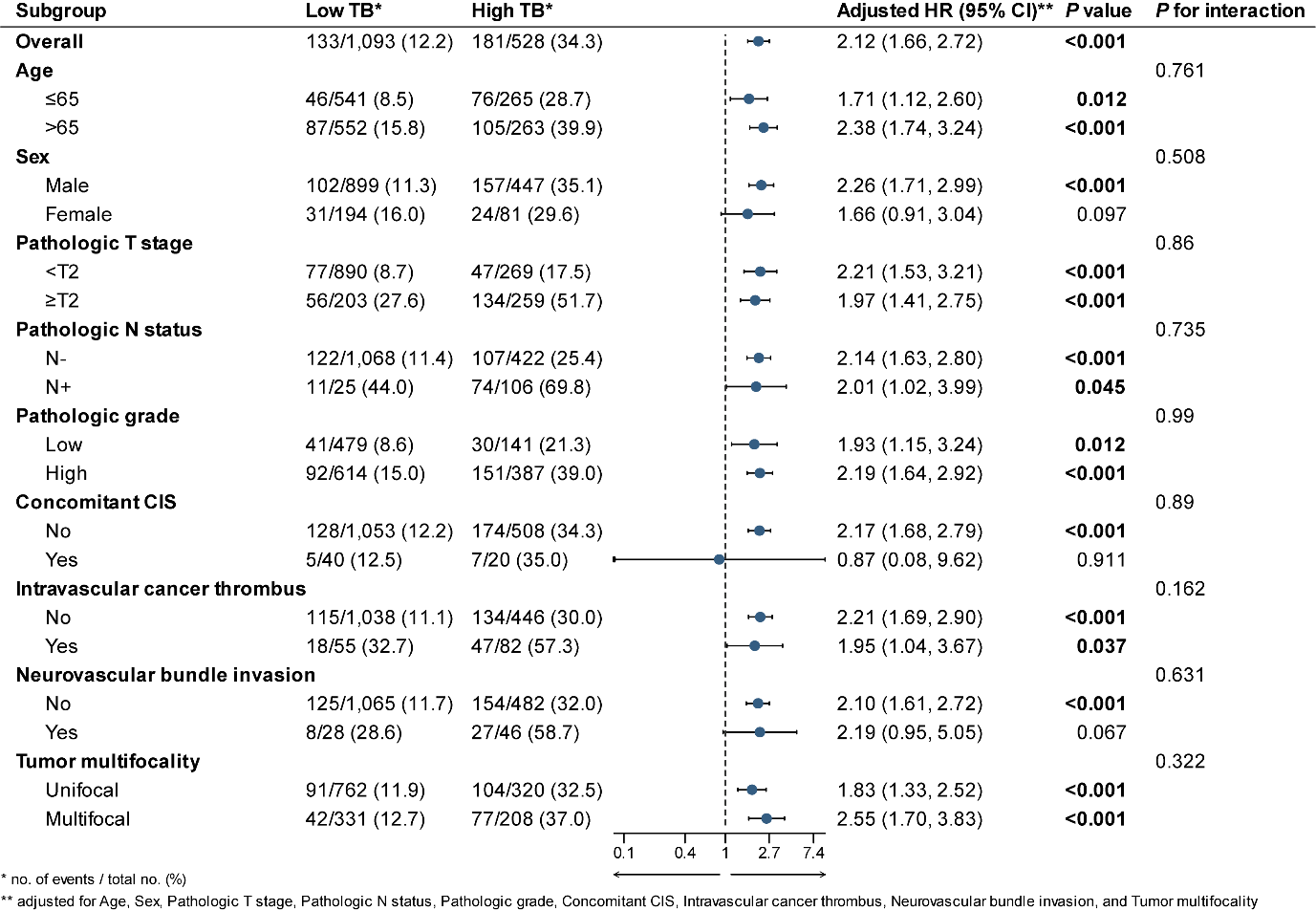
**

**Figure S3. Subgroup analysis of overall survival in the combined cohort.**

HRs and *P*-values were estimated using the log-rank test. 95% CI, 95% confidence interval; CIS, carcinoma in situ; HR, hazard ratio; LVI, lymphovascular invasion; PNI, perineural invasion; TB, tumour budding.

| 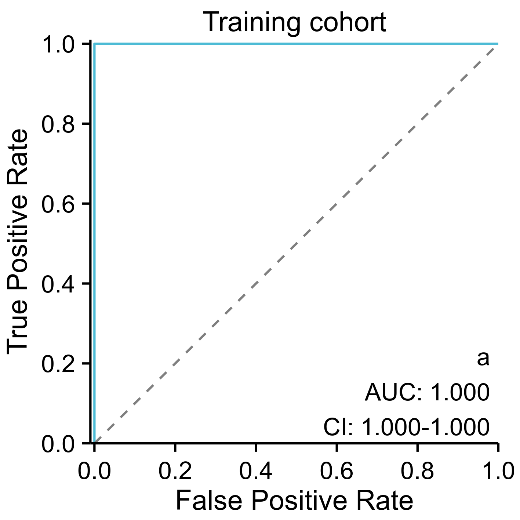 | 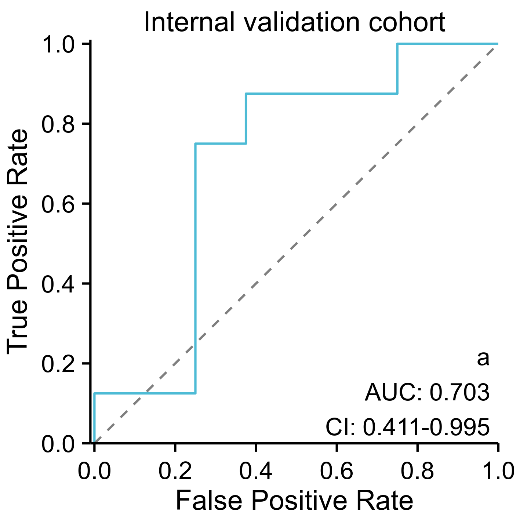 |
| --- | --- |
| **Figure S4. AUCs and CIs of results obtained using the NACI response prediction model in patients underwent NACI.**  AUC, area under the curve; CI, confidence interval; NACI: neoadjuvant chemoimmunotherapy. | |

**
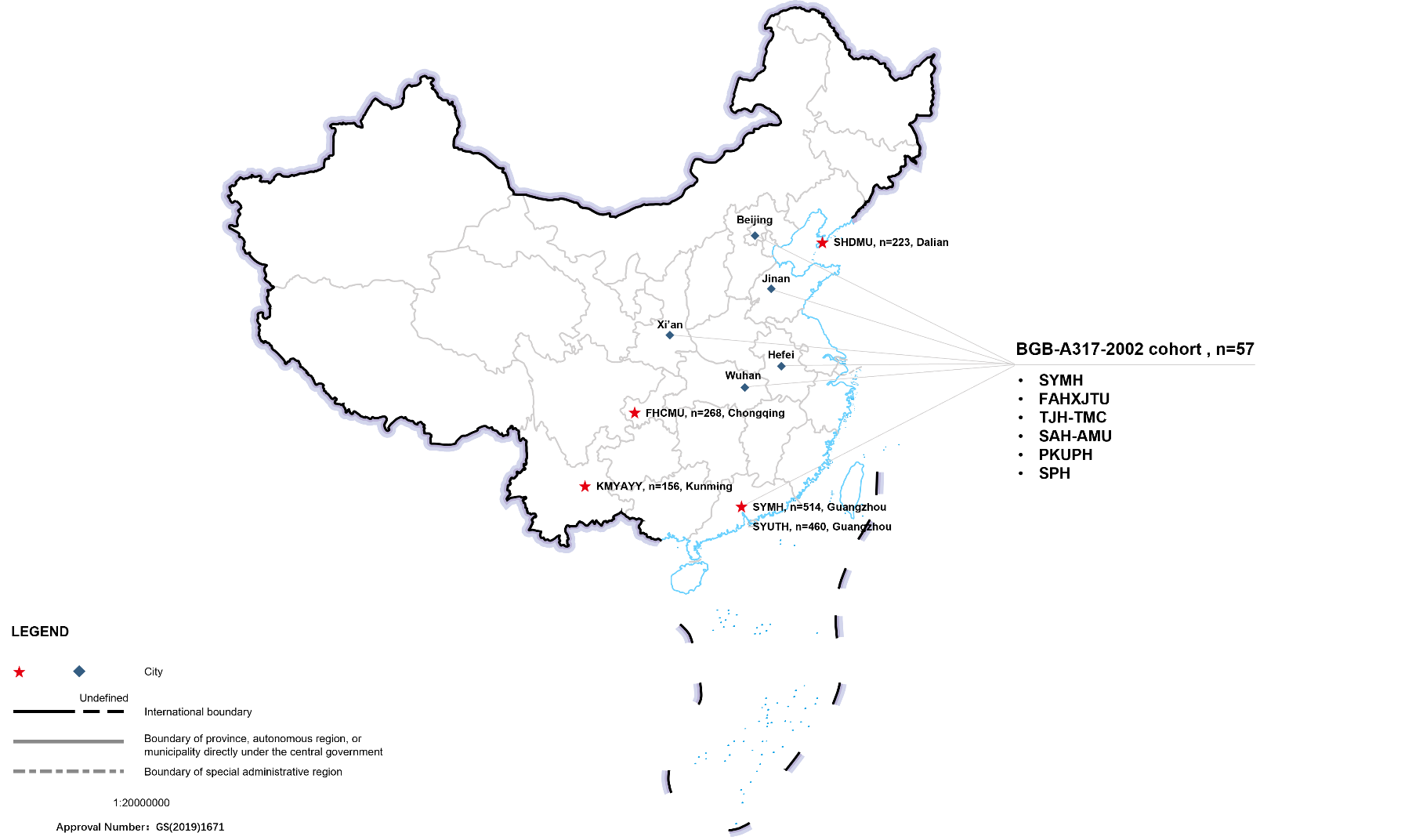
**

**Figure S5. Geographic distribution of the included hospitals in China and the number of patients enrolled from each hospital.**

FAHXJTH, First Affiliated Hospital of Xi’an Jiaotong University; FHCMU, First Affiliated Hospital of Chongqing Medical University; KMYAYY, Yan’an Hospital Affiliated to Kunming Medical University; PKUPH, Peking University People’s Hospital; SAH-AMU, The Second Hospital of Anhui Medical University; SPH, Shandong Provincial Hospital; SHDMU, Second Hospital of Dalian Medical University; SYMH, Sun Yat-sen Memorial Hospital; SYUTH, Third Affiliated Hospital of Sun Yat-sen University; TJH-TMC, Tongji Hospital, Tongji Medical College

**
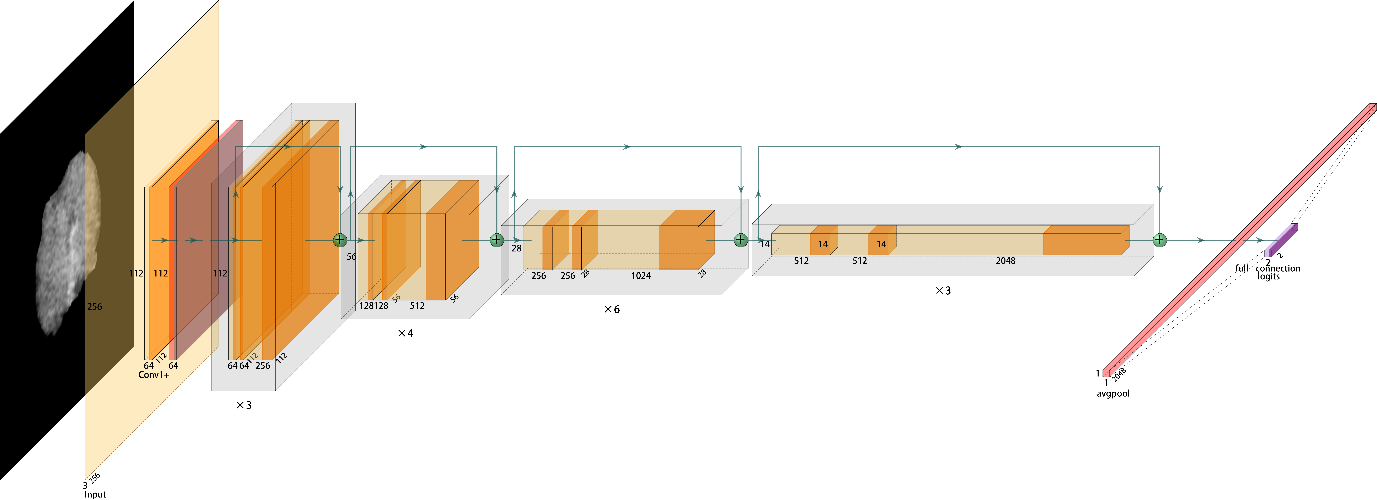
**

**Figure S6. Architecture of the deep learning model (ResNet-50) used for tumour budding prediction.**

**Supplemental Tables**

| **Table S1. Clinicopathological characteristics of patients in the multi-institutional cohorts.** | |
| --- | --- |
| **Characteristics** | **Overall** |
| **N** | 2113 |
| **Age, median (IQR), years** | 66.0 (59.0, 74.0) |
| **Sex** |  |
| Men (%) | 1724 (81.6) |
| Women (%) | 389 (18.4) |
| **T stage** |  |
| ＜T2 (%) | 1170 (55.4) |
| ≥T2 (%) | 943 (44.6) |
| **N status** |  |
| N- (%) | 1823 (86.3) |
| N+ (%) | 290 (13.7) |
| **Grade** |  |
| Low (%) | 643 (31.3) |
| High (%) | 1413 (68.7) |
| **TB status** |  |
| Low TB (%) | 1343 (63.6) |
| High TB (%) | 770 (36.4) |
| IQR, interquartile range; TB, tumour budding. | |

| **Table S2. Clinicopathological characteristics of patients in the BGB-A317-2002 cohort.** | |
| --- | --- |
| **Characteristics** | **BGB-A317-2002 cohort** |
| **N** | 57 |
| **Age, median (IQR), years** | 64·0 (59·0, 68·0) |
| **Sex** |  |
| Men (%) | 49 (86) |
| Women (%) | 8 (14) |
| **TB status** |  |
| Low (%) | 29(50·9) |
| High (%) | 28(49·1) |
| **T stage** |  |
| T2 (%) | 36 (63·2) |
| T3 (%) | 17 (29·8) |
| T4 (%) | 4 (7) |
| **N status** |  |
| N- (%) | 48 (84·2) |
| N+ (%) | 9 (15·8) |
| **Response** |  |
| non-pCR (%) | 28 (49·1) |
| pCR (%) | 29 (50·9) |
| IQR, interquartile range; pCR, pathological complete response; TB, tumour budding. | |

**Table S3. Clinicopathological characteristics of patients in the SYMH and external cohorts ^a^.**

| **Characteristics** | **Total** | **SYMH cohort** | **External cohort 1** | **External cohort 2** | ***P* value** |
| --- | --- | --- | --- | --- | --- |
| **N** | **1621** | **514** | **460** | **647** |  |
| **Age, median (IQR), years** | 66·0 (58·0, 74·0) | 64·0 (57·0, 71·8) | 65·0 (56·0, 72·0) | 68·0 (61·0, 76·0) | **< 0·001** |
| **Sex** |  |  |  |  | 0·17 |
| Men (%) | 1346 (83) | 440 (85·6) | 377 (82) | 529 (81·8) |  |
| Women (%) | 275 (17) | 74 (14·4) | 83 (18) | 118 (18·2) |  |
| **T stage** |  |  |  |  | **< 0·001** |
| <T2 (%) | 1159 (71·5) | 329 (64) | 356 (77·4) | 474 (73·3) |  |
| ≥T2 (%) | 462 (28·5) | 185 (36) | 104 (22·6) | 173 (26·7) |  |
| **N status** |  |  |  |  | **< 0·001** |
| N- (%) | 1490 (91·9) | 455 (88·5) | 435 (94·6) | 600 (92·7) |  |
| N+ (%) | 131 (8·1) | 59 (11·5) | 25 (5·4) | 47 (7·3) |  |
| **Grade** |  |  |  |  | **< 0·001** |
| Low (%) | 620 (38·2) | 127 (24·7) | 221 (48) | 272 (42) |  |
| High (%) | 1001 (61·8) | 387 (75·3) | 239 (52) | 375 (58) |  |
| **TB status** |  |  |  |  | **< 0·001** |
| Low (%) | 1093 (67·4) | 296 (57·6) | 344 (74·8) | 453 (70) |  |
| High (%) | 528 (32·6) | 218 (42·4) | 116 (25·2) | 194 (30) |  |
| **Concomitant CIS** |  |  |  |  | 0·52 |
| No (%) | 1561 (96·3) | 496 (96·5) | 446 (97) | 619 (95·7) |  |
| Yes (%) | 60 (3·7) | 18 (3·5) | 14 (3) | 28 (4·3) |  |
| **Lymphovascular invasion** |  |  |  |  | 0·07 |
| No (%) | 1484 (91·5) | 459 (89·3) | 429 (93·3) | 596 (92·1) |  |
| Yes (%) | 137 (8·5) | 55 (10·7) | 31 (6·7) | 51 (7·9) |  |
| **Perineural invasion** |  |  |  |  | 0·62 |
| No (%) | 1547 (95·4) | 494 (96·1) | 439 (95·4) | 614 (94·9) |  |
| Yes (%) | 74 (4·6) | 20 (3·9) | 21 (4·6) | 33 (5·1) |  |
| **Tumour multifocality** |  |  |  |  | **< 0·001** |
| Unifocal (%) | 1082 (66·7) | 265 (51·6) | 365 (79·3) | 452 (69·9) |  |
| Multifocal (%) | 539 (33·3) | 249 (48·4) | 95 (20·7) | 195 (30·1) |  |
| Bold values are statistically significant.  ^a^Data are presented as numbers (percentages) of patients unless otherwise indicated.  CIS, carcinoma in situ; IQR, interquartile range; SYMH, Sun Yat-sen Memorial Hospital; TB, tumour budding. | | | | | |

| **Table S4. Clinicopathological characteristics of patients in the TCGA and NACI real-world cohorts.** | | |
| --- | --- | --- |
| **Characteristics** | **TCGA cohort** | **NACI real-world cohort** |
| **N** | 327 | 108 |
| **Age, median (IQR), years** | 69·0 (60·0, 76·5) | 64·0 (57·0, 70·0) |
| **Sex** |  |  |
| Men (%) | 239 (73·1) | 90 (83·3) |
| Women (%) | 88 (26·9) | 18 (16·7) |
| **TB status** |  |  |
| Low (%) | 171 (52·3) | 50 (46·3) |
| High (%) | 156 (47·7) | 58 (53·7) |
| **T stage** |  |  |
| <T2 (%) | 1 (0·3) | 10 (9·3) |
| ≥T2 (%) | 326 (99·7) | 98 (90·7) |
| **N status** |  |  |
| N- (%) | 210 (64·2) | 75 (69·4) |
| N+ (%) | 117 (35·8) | 33 (30·6) |
| **Grade** |  |  |
| Low (%) | 17 (5·2) | 6 (5·6) |
| High (%) | 310 (94·8) | 102 (94·4) |
| IQR, interquartile range; NCAI, neoadjuvant chemoimmunotherapy; TB, tumour budding; TCGA, The Cancer Genome Atlas database. | | |

**Table S5. Time-dependent Cox regression analysis of the association between TB and OS in the combined cohort.**

| **Characteristics** | **Total** | **Univariate analysis** | |  | **Multivariate analysis** | |
| --- | --- | --- | --- | --- | --- | --- |
|  |  | **HR (95% CI)** | ***P*-value** |  | **HR (95% CI)** | ***P*-value** |
| **Age** | 1,621 | 1·035 (1·025 - 1·045) | **< 0·001** |  | 1·032 (1·022 - 1·042) | **< 0·001** |
| **Sex** | 1,621 |  |  |  |  |  |
| Men | 1,346 | Reference |  |  |  |  |
| Women | 275 | 0·886 (0·662 - 1·185) | 0·41 |  |  |  |
| **T stage** | 1,621 |  |  |  |  |  |
| <T2 | 1,159 | Reference |  |  | Reference |  |
| ≥T2 | 462 | 5·576 (4·438 - 7·005) | **< 0·001** |  | 2·925 (2·181 - 3·921) | **< 0·001** |
| **N status** | 1,621 |  |  |  |  |  |
| N- | 1,490 | Reference |  |  | Reference |  |
| N+ | 131 | 7·359 (5·707 - 9·489) | **< 0·001** |  | 2·240 (1·653 - 3·037) | **< 0·001** |
| **Grade** | 1,621 |  |  |  |  |  |
| Low | 620 | Reference |  |  | Reference |  |
| High | 1,001 | 2·831 (2·169 - 3·695) | **< 0·001** |  | 1·214 (0·892 - 1·652) | 0·22 |
| **TB status** | 1,621 |  |  |  |  |  |
| Low | 1,093 | Reference |  |  | Reference |  |
| High | 528 | 0·290 (0·232 - 0·363) | **< 0·001** |  | 0·487 (0·380 - 0·623) | **< 0·001** |
| **Concomitant CIS** | 1,621 |  |  |  |  |  |
| No | 1,561 | Reference |  |  |  |  |
| Yes | 60 | 1·556 (0·873 - 2·773) | 0·13 |  |  |  |
| **Lymphovascular invasion** | 1,621 |  |  |  |  |  |
| No | 1,484 | Reference |  |  | Reference |  |
| Yes | 137 | 3·822 (2·903 - 5·032) | **< 0·001** |  | 1·220 (0·873 - 1·705) | 0·24 |
| **Perineural invasion** | 1,621 |  |  |  |  |  |
| No | 1,547 | Reference |  |  | Reference |  |
| Yes | 74 | 3·768 (2·647 - 5·365) | **< 0·001** |  | 0·989 (0·661 - 1·478) | 0·96 |
| **Tumour multifocality** | 1,621 |  |  |  |  |  |
| Unifocal | 1,082 | Reference |  |  | Reference |  |
| Multifocal | 539 | 1·274 (1·015 - 1·601) | **0·04** |  | 1·050 (0·834 - 1·321) | 0·68 |
| CIS, carcinoma in situ; HR, hazard ratio; OS, overall survival; TB, tumour budding; 95% CI, 95% confidence interval. | | | | | | |

**Table S6. Time-dependent Cox regression analysis of the association between TB and OS in the SYMH cohort.**

| **Characteristics** | **Total** | **Univariate analysis** | |  | **Multivariate analysis** | |
| --- | --- | --- | --- | --- | --- | --- |
|  |  | **HR (95% CI)** | ***P*-value** |  | **HR (95% CI)** | ***P*-value** |
| **Age^a^** | 514 | 1·024 (1·009 - 1·039) | **0·001** |  | 1·023 (1·007 - 1·039) | **0·00** |
| **Sex** | 514 |  |  |  |  |  |
| Men | 440 | Reference |  |  |  |  |
| Women | 74 | 1·267 (0·794 - 2·022) | 0·32 |  |  |  |
| **T stage^a^** | 514 |  |  |  |  |  |
| <T2 | 329 | Reference |  |  | Reference |  |
| ≥T2 | 185 | 5·593 (3·873 - 8·075) | **< 0·001** |  | 3·313 (2·146 - 5·116) | **< 0·001** |
| **N status^a^** | 514 |  |  |  |  |  |
| N- | 455 | Reference |  |  | Reference |  |
| N+ | 59 | 5·545 (3·818 - 8·054) | **< 0·001** |  | 1·789 (1·137 - 2·815) | **0·01** |
| **Grade** | 514 |  |  |  |  |  |
| Low | 127 | Reference |  |  | Reference |  |
| High | 387 | 2·359 (1·507 - 3·693) | **< 0·001** |  | 1·148 (0·703 - 1·875) | 0·58 |
| **TB status** | 514 |  |  |  |  |  |
| Low | 296 | Reference |  |  | Reference |  |
| High | 218 | 3·409 (2·361 - 4·922) | **< 0·001** |  | 2·237 (1·515 - 3·304) | **< 0·001** |
| **Concomitant CIS** | 514 |  |  |  |  |  |
| No | 18 | Reference |  |  |  |  |
| Yes | 496 | 1·688 (0·689 - 4·134) | 0·25 |  |  |  |
| **Lymphovascular invasion** | 514 |  |  |  |  |  |
| No | 459 | Reference |  |  | Reference |  |
| Yes | 55 | 2·901 (1·885 - 4·463) | **< 0·001** |  | 1·012 (0·599 - 1·710) | 0·97 |
| **Perineural invasion^a^** | 514 |  |  |  |  |  |
| No | 494 | Reference |  |  | Reference |  |
| Yes | 20 | 4·347 (2·337 - 8·087) | **< 0·001** |  | 1·543 (0·755 - 3·151) | 0·23 |
| **Tumour multifocality** | 514 |  |  |  |  |  |
| Unifocal | 265 | Reference |  |  |  |  |
| Multifocal | 249 | 1·022 (0·727 - 1·436) | 0·90 |  |  |  |
| ^a^ Multiplicative interaction terms between variables that violated the PH assumption and the natural logarithm of time were used to perform time-dependent Cox regression analysis. HRs should not be interpreted as independent associations but rather to predict the risk of overall survival.  CIS, carcinoma in situ; HR, hazard ratio; OS, overall survival; SYMH, Sun Yat-sen Memorial Hospital; TB, tumour budding; 95% CI, 95% confidence interval. | | | | | | |

**Table S7. Time-dependent Cox regression analysis of the association between TB and OS in external cohort 1.**

| **Characteristics** | **Total** | **Univariate analysis** | |  | **Multivariate analysis** | |
| --- | --- | --- | --- | --- | --- | --- |
|  |  | **HR (95% CI)** | ***P*-value** |  | **HR (95% CI)** | ***P*-value** |
| **Age** | 460 | 1·028 (1·012 - 1·045) | **< 0·001** |  | 1·029 (1·012 - 1·046) | **< 0·001** |
| **Sex** | 460 |  |  |  |  |  |
| Men | 83 | Reference |  |  |  |  |
| Women | 377 | 1·318 (0·776 - 2·238) | 0·31 |  |  |  |
| **T stage** | 460 |  |  |  |  |  |
| <T2 | 356 | Reference |  |  | Reference |  |
| ≥T2 | 104 | 6·103 (4·183 - 8·905) | **< 0·001** |  | 2·580 (1·552 - 4·290) | **< 0·001** |
| **N status^a^** | 460 |  |  |  |  |  |
| N- | 435 | Reference |  |  | Reference |  |
| N+ | 25 | 12·360 (7·287 - 20·965) | **< 0·001** |  | 3·295 (1·762 - 6·163) | **< 0·001** |
| **Grade** | 460 |  |  |  |  |  |
| Low | 221 | Reference |  |  | Reference |  |
| High | 239 | 4·669 (2·922 - 7·459) | **< 0·001** |  | 2·165 (1·246 - 3·765) | **0·01** |
| **TB status** | 460 |  |  |  |  |  |
| Low | 344 | Reference |  |  | Reference |  |
| High | 116 | 3·036 (2·084 - 4·425) | **< 0·001** |  | 1·629 (1·033 - 2·570) | **0·04** |
| **Concomitant CIS** | 460 |  |  |  |  |  |
| No | 446 | Reference |  |  |  |  |
| Yes | 14 | 0·842 (0·267 - 2·652) | 0·77 |  |  |  |
| **Lymphovascular invasion** | 460 |  |  |  |  |  |
| No | 429 | Reference |  |  | Reference |  |
| Yes | 31 | 5·014 (3·090 - 8·136) | **< 0·001** |  | 1·499 (0·859 - 2·619) | 0·15 |
| **Perineural invasion** | 460 |  |  |  |  |  |
| No | 439 | Reference |  |  | Reference |  |
| Yes | 21 | 3·508 (1·873 - 6·568) | **< 0·001** |  | 0·922 (0·466 - 1·824) | 0·82 |
| **Tumour multifocality** | 460 |  |  |  |  |  |
| Unifocal | 365 | Reference |  |  | Reference |  |
| Multifocal | 95 | 1·519 (1·003 - 2·300) | **0·05** |  | 1·332 (0·870 - 2·040) | 0·19 |
| ^a^ Multiplicative interaction terms between variables that violated the PH assumption and the natural logarithm of time were used to perform time-dependent Cox regression analysis. HRs should not be interpreted as independent associations but rather to predict the risk of overall survival.  CIS, carcinoma in situ; HR, hazard ratio; OS, overall survival; TB, tumour budding; 95% CI, 95% confidence interval. | | | | | | |

**Table S8. Time-dependent Cox regression analysis of the association between TB and OS in external cohort 2.**

| **Characteristics** | **Total** | **Univariate analysis** | |  | **Multivariate analysis** | |
| --- | --- | --- | --- | --- | --- | --- |
|  |  | **HR (95% CI)** | ***P*-value** |  | **HR (95% CI)** | ***P*-value** |
| **Age** | 647 | 1·083 (1·055 - 1·111) | **< 0·001** |  | 1·075 (1·046 - 1·104) | **< 0·001** |
| **Sex** | 647 |  |  |  |  |  |
| Men | 529 | Reference |  |  | Reference |  |
| Women | 118 | 1·799 (1·047 - 3·091) | **0·03** |  | 2·725 (1·525 - 4·869) | **< 0·001** |
| **T stage** | 647 |  |  |  |  |  |
| <T2 | 474 | Reference |  |  | Reference |  |
| ≥T2 | 173 | 4·741 (2·933 - 7·663) | **< 0·001** |  | 2·957 (1·515 - 5·773) | **0·00** |
| **N status** | 647 |  |  |  |  |  |
| N- | 600 | Reference |  |  | Reference |  |
| N+ | 47 | 7·908 (4·765 - 13·123) | **< 0·001** |  | 2·554 (1·333 - 4·894) | **0·01** |
| **Grade^a^** | 647 |  |  |  |  |  |
| Low | 272 | Reference |  |  | Reference |  |
| High | 375 | 1·755 (1·067 - 2·886) | **0·03** |  | 0·605 (0·316 - 1·155) | 0·12 |
| **TB status** | 647 |  |  |  |  |  |
| Low | 453 | Reference |  |  | Reference |  |
| High | 194 | 4·186 (2·595 - 6·753) | **< 0·001** |  | 2·498 (1·458 - 4·281) | **< 0·001** |
| **Concomitant CIS** | 647 |  |  |  |  |  |
| No | 619 | Reference |  |  | Reference |  |
| Yes | 28 | 4·684 (1·667 - 13·164) | **0·00** |  | 2·145 (0·687 - 6·703) | 0·19 |
| **Lymphovascular invasion** | 647 |  |  |  |  |  |
| No | 596 | Reference |  |  | Reference |  |
| Yes | 51 | 4·028 (2·350 - 6·905) | **< 0·001** |  | 1·236 (0·520 - 2·936) | 0·63 |
| **Perineural invasion^a^** | 647 |  |  |  |  |  |
| No | 614 | Reference |  |  | Reference |  |
| Yes | 33 | 3·504 (1·904 - 6·448) | **< 0·001** |  | 0·954 (0·402 - 2·268) | 0·92 |
| **Tumour multifocality** | 647 |  |  |  |  |  |
| Unifocal | 452 | Reference |  |  |  |  |
| Multifocal | 195 | 1·254 (0·754 - 2·087) | 0·38 |  |  |  |
| ^a^ Multiplicative interaction terms between variables that violated the PH assumption and the natural logarithm of time were used to perform time-dependent Cox regression analysis. HRs should not be interpreted as independent associations but rather to predict the risk of overall survival.  CIS, carcinoma in situ; HR, hazard ratio; OS, overall survival; TB, tumour budding; 95% CI, 95% confidence interval. | | | | | | |

**Table S9. Clinicopathological characteristics of patients in the high- and low-TB groups in the SYMH and external cohorts.**

| **Characteristics** | **SYMH cohort** | | | **External cohort 1** | | | **External cohort 2** | | |
| --- | --- | --- | --- | --- | --- | --- | --- | --- | --- |
|  | **High TB** | **Low TB** | ***P*-value** | **High TB** | **Low TB** | ***P*-value** | **High TB** | **Low TB** | ***P*-value** |
| **N** | 218 | 296 |  | 116 | 344 |  | 194 | 453 |  |
| **Age, median (IQR), years** | 67·0 (56·0, 71·0) | 64·5 (58·0, 72·0) | 0·53 | 65·5 (56·0, 74·0) | 64·0 (56·0, 72·0) | 0·44 | 70·0 (63·0, 78·0) | 68·0 (60·0, 75·0) | **0·06** |
| **Sex** |  |  | 0·92 |  |  | **0·03** |  |  | 0·72 |
| Men | 187 (85·8) | 253 (85·5) |  | 103 (88·8) | 274 (79·7) |  | 157 (80·9) | 372 (82·1) |  |
| Women | 31 (14·2) | 43 (14·5) |  | 13 (11·2) | 70 (20·3) |  | 37 (19·1) | 81 (17·9) |  |
| **T stage** |  |  | **< 0·001** |  |  | **< 0·001** |  |  | **< 0·001** |
| <T2 | 110 (50·5) | 219 (74) |  | 60 (51·7) | 296 (86) |  | 99 (51) | 375 (82·8) |  |
| ≥T2 | 108 (49·5) | 77 (26) |  | 56 (48·3) | 48 (14) |  | 95 (49) | 78 (17·2) |  |
| **N status** |  |  | **< 0·001** |  |  | **< 0·001** |  |  | **< 0·001** |
| N- | 168 (77·1) | 287 (97·0) |  | 94 (81) | 341 (99·1) |  | 160 (82·5) | 440 (97·1) |  |
| N+ | 50 (22·9) | 9 (3·0) |  | 22 (19) | 3 (0·9) |  | 34 (17·5) | 13 (2·9) |  |
| **Grade** |  |  | **0·01** |  |  | **< 0·001** |  |  | **0·001** |
| Low | 42 (19·3) | 85 (28·7) |  | 36 (31) | 185 (53·8) |  | 63 (32·5) | 209 (46·1) |  |
| High | 176 (80·7) | 211 (71·3) |  | 80 (69) | 159 (46·2) |  | 131 (67·5) | 244 (53·9) |  |
| **Concomitant CIS** |  |  | 0·51 |  |  | 1·00 |  |  | 0·56 |
| No | 209 (95·9) | 287 (97) |  | 112 (96·6) | 334 (97·1) |  | 187 (96·4) | 432 (95·4) |  |
| Yes | 9 (4·1) | 9 (3) |  | 4 (3·4) | 10 (2·9) |  | 7 (3·6) | 21 (4·6) |  |
| **Lymphovascular invasion** |  |  | **< 0·001** |  |  | **0·002** |  |  | **< 0·001** |
| No | 182 (83·5) | 277 (93·6) |  | 101 (87·1) | 328 (95·3) |  | 163 (84) | 433 (95·6) |  |
| Yes | 36 (16·5) | 19 (6·4) |  | 15 (12·9) | 16 (4·7) |  | 31 (16) | 20 (4·4) |  |
| **Perineural invasion** |  |  | **0·04** |  |  | **< 0·001** |  |  | **< 0·001** |
| No | 205 (94) | 289 (97·6) |  | 102 (87·9) | 337 (98) |  | 175 (90·2) | 439 (96·9) |  |
| Yes | 13 (6) | 7 (2·4) |  | 14 (12·1) | 7 (2) |  | 19 (9·8) | 14 (3·1) |  |
| **Tumour multifocality** |  |  | 0·06 |  |  | 0·42 |  |  | 0·22 |
| Unifocal | 102 (46·8) | 163 (55·1) |  | 89 (76·7) | 276 (80·2) |  | 129 (66·5) | 323 (71·3) |  |
| Multifocal | 116 (53·2) | 133 (44·9) |  | 27 (23·3) | 68 (19·8) |  | 65 (33·5) | 130 (28·7) |  |
| CIS, carcinoma in situ; HR, hazard ratio; IQR, interquartile range; SYMH, Sun Yat-sen Memorial Hospital; TB, tumour budding. | | | | | | | | | |

**Table S10. Clinicopathological characteristics of patients in the high- and low-TB groups in the TCGA cohort ^a^.**

| **Characteristics** | **Low TB** | **High TB** | ***P*-value** |
| --- | --- | --- | --- |
| **N** | 171 | 156 |  |
| **Age, median (IQR), years** | 69·0 (60·0, 75·0) | 69·0 (61·0, 78·0) | 0·31 |
| **Sex** |  |  | 1·00 |
| Men (%) | 125 (73·1) | 114 (73·1) |  |
| Women (%) | 46 (26·9) | 42 (26·9) |  |
| **T stage** |  |  | 1·00 |
| <T2 (%) | 1 (0·6) | 0 (0) |  |
| ≥T2 (%) | 170 (99·4) | 156 (100) |  |
| **N status** |  |  | **<0·001** |
| N- (%) | 156 (91·2) | 54 (34·6) |  |
| N+ (%) | 15 (8·8) | 102 (65·4) |  |
| **Grade** |  |  | **<0·001** |
| Low (%) | 16 (9·4) | 1 (0·6) |  |
| High (%) | 155 (90·6) | 155 (99·4) |  |
| ^a^ Data are presented as numbers (percentages) of patients unless otherwise indicated.  IQR, interquartile range; TB, tumour budding; TCGA, The Cancer Genome Atlas database. | | | |

| **Table S11. Clinicopathological characteristics of patients in the high- and low-TB groups in the BGB-A317-2002 cohort ^a^.** | | | |
| --- | --- | --- | --- |
| **Characteristics** | **Low TB** | **High TB** | ***P*-value** |
| **N** | 29 | 28 |  |
| **Age, median (IQR), years** | 63·0 (60·0, 67·0) | 65·5 (58·5, 67·0) | 0·46 |
| **Sex** |  |  | 0·28 |
| Men (%) | 23 (79·3) | 26 (92·9) |  |
| Women (%) | 6 (20·7) | 2 (7·1) |  |
| **T stage** |  |  | 0·62 |
| T2 (%) | 20 (69) | 16 (57·1) |  |
| T3 (%) | 7 (24·1) | 10 (35·7) |  |
| T4 (%) | 2 (6·9) | 2 (7·1) |  |
| **N status** |  |  | 0·13 |
| N- (%) | 27 (93·1) | 21 (75) |  |
| N+ (%) | 2 (6·9) | 7 (25) |  |
| **Response** |  |  | **< 0·001** |
| non-pCR (%) | 6 (20·7) | 22 (78·6) |  |
| pCR (%) | 23 (79·3) | 6 (21·4) |  |
| ^a^ Data are presented as numbers (percentages) of patients unless otherwise indicated.  IQR, interquartile range; pCR, pathological complete response; SD, standard deviation; TB, tumour budding. | | | |

| **Table S12. Clinicopathological characteristics of patients in the high- and low-TB groups in the NACI real-world cohort** **^a^.** | | | |
| --- | --- | --- | --- |
| **Characteristics** | **Low TB** | **High TB** | ***P*-value** |
| **N** | 50 | 58 |  |
| **Age, median (IQR), years** | 65·0 (59·25, 70·0) | 63·0 (57·0, 69·0) | 0·73 |
| **Sex** |  |  | 0·39 |
| Men (%) | 40 (80) | 50 (86·2) |  |
| Women (%) | 10 (20) | 8 (13·8) |  |
| **T stage** |  |  | 1·00 |
| <T2 (%) | 5 (10) | 5 (8·6) |  |
| ≥T2 (%) | 45 (90) | 53 (91·4) |  |
| **N status** |  |  | 0·17 |
| N- (%) | 38 (76) | 37 (63·8) |  |
| N+ (%) | 12 (24) | 21 (36·2) |  |
| **Grade** |  |  | 1·00 |
| Low (%) | 3 (6) | 3 (5·2) |  |
| High (%) | 47 (94) | 55 (94·8) |  |
| **Response** |  |  | **< 0·001** |
| pCR (%) | 41 (82) | 8 (13·8) |  |
| non-pCR (%) | 9 (18) | 50 (86·2) |  |
| ^a^ Data are presented as numbers (percentages) of patients unless otherwise indicated.  IQR, interquartile range; NACI, neoadjuvant chemoimmunotherapy; pCR, pathological complete response; TB, tumour budding. | | | |

**Table S13. Time-dependent Cox regression analysis of the association between the predicted TB status and OS in the combined cohort.**

| **Characteristics** | **Total** | **Univariate analysis** | |  | **Multivariate analysis** | |
| --- | --- | --- | --- | --- | --- | --- |
|  |  | **HR (95% CI)** | ***P*-value** |  | **HR (95% CI)** | ***P*-value** |
| **Age** | 1,094 | 1·030 (1·019 - 1·041) | **< 0·001** |  | 1·028 (1·016 - 1·040) | **< 0·001** |
| **Sex** | 1,094 |  |  |  |  |  |
| Men | 906 | Reference |  |  |  |  |
| Women | 188 | 1·061 (0·758 - 1·485) | 0·73 |  |  |  |
| **T stage** | 1,094 |  |  |  |  |  |
| <T2 | 810 | Reference |  |  | Reference |  |
| ≥T2 | 284 | 5·164 (4·004 - 6·660) | **< 0·001** |  | 2·615 (1·874 - 3·648) | **< 0·001** |
| **N status^a^** | 1,094 |  |  |  |  |  |
| N- | 996 | Reference |  |  | Reference |  |
| N+ | 98 | 7·626 (5·734 - 10·142) | **< 0·001** |  | 2·712 (1·905 - 3·863) | **< 0·001** |
| **Grade** | 1,094 |  |  |  |  |  |
| Low | 423 | Reference |  |  | Reference |  |
| High | 671 | 2·504 (1·869 - 3·356) | **< 0·001** |  | 1·127 (0·807 - 1·573) | 0·48 |
| **Predicted TB status** | 1,094 |  |  |  |  |  |
| Low | 768 | Reference |  |  | Reference |  |
| High | 326 | 2·951 (2·292 – 3·799) | **< 0·001** |  | 1·771 (1·353 - 2·318) | **< 0·001** |
| **Concomitant CIS** | 1,094 |  |  |  |  |  |
| No | 1,048 | Reference |  |  |  |  |
| Yes | 46 | 1·532 (0·813 - 2·887) | 0·19 |  |  |  |
| **Lymphovascular invasion** | 1,094 |  |  |  |  |  |
| No | 996 | Reference |  |  | Reference |  |
| Yes | 98 | 3·855 (2·837 - 5·238) | **< 0·001** |  | 1·153 (0·786 - 1·692) | 0·47 |
| **Perineural invasion** | 1,094 |  |  |  |  |  |
| No | 1,040 | Reference |  |  | Reference |  |
| Yes | 54 | 4·388 (2·983 - 6·456) | **< 0·001** |  | 1·427 (0·916 - 2·224) | 0·12 |
| **Tumour multifocality** | 1,094 |  |  |  |  |  |
| Unifocal | 724 | Reference |  |  |  |  |
| Multifocal | 370 | 1·233 (0·953 - 1·596) | 0·11 |  |  |  |
| ^a^ Multiplicative interaction terms between variables that violated the PH assumption and the natural logarithm of time were used to perform time-dependent Cox regression analysis. HRs should not be interpreted as independent associations but rather to predict the risk of overall survival.  TB, tumour budding; HR, hazard ratio; OS, overall survival; CIS, carcinoma in situ; 95% CI, 95% confidence interval. | | | | | | |

| **Table S14. Performance of the NACI response prediction model in patients underwent NACI.** | | | | | |
| --- | --- | --- | --- | --- | --- |
|  | **Over Accuracy**  **(%)** | **Sensitivity**  **(95% CI)** | **Specificity**  **(95% Cl)** | **PPV**  **(95% Cl)** | **NPV**  **(95% Cl)** |
| **Training cohort** | 91.17 | 81.25 | 100 | 100 | 85.71 |
| **Internal validation cohort** | 50 | 12.5 | 87.5 | 35.35 | 13.36 |
| AUC, area under the curve; 95% CI, 95% confidence interval; NACI, neoadjuvant chemoimmunotherapy; NPV, negative predictive value; PPV, positive predictive value. | | | | | |
